# Application of modified Yamane technique in intrascleral intraocular lens fixation combined with or without iris reconstruction

**DOI:** 10.1186/s12886-024-03493-8

**Published:** 2024-06-05

**Authors:** Xiaofang Wang, Mengru Su, Yushan Li, Hairong Xie, Xinghong Sun, Feng Jiang

**Affiliations:** Department of Ophthalmology, Nanjing Drum Tower Hospital, The Affiliated Hospital of Nanjing University Medical School, Nanjing, Jiangsu China

**Keywords:** Modified yamane technique, Intrascleral intraocular lens fixation, Iris reconstruction

## Abstract

**Aims:**

To explore the application and long-term clinical effects of modified Yamane technique in intrascleral intraocular lens (IOL) fixation combined with or without iris reconstruction.

**Settings and design:**

The data of patients receiving IOL fixation with modified Yamane technique in an ophthalmology department between December 2021 and August 2023 were analyzed retrospectively. The longest follow-up duration was > 12 months.

**Methods and material:**

The trailing haptic was fixed with the needle before the leading haptic. The silicone haptic stoppers were used to stabilize the IOL when iris reconstruction was combined. Preoperative and postoperative best-corrected visual acuity (BCVA), corneal endothelial cells (CECs), postoperative intraocular pressure (IOP), surgical indications and methods, and postoperative complications were recorded. Anterior segment optical coherence tomography (OCT) was used to evaluate IOL decentration and tilt. The paired sample t-test or Wilcoxon rank sum test were used to compare the results of the same index before and after the operation.

**Results:**

Twelve patients (12 eyes) were included in this cohort. There were 1 case of IOL dislocation, eight cases of lens dislocation or subluxation, and three cases of aphakia. Traumatic lens dislocation was the main cause of aphakia. Primary lens extraction was performed in previous surgeries, and all three were combined with pars plana vitrectomy (PPV). Four of 12 patients underwent IOL fixation and iris reconstruction. The mean age of participants was 63 ± 10.61 years. The mean BCVA increased from 0.89 ± 0.72 logMAR to 0.39 ± 0.56 logMAR at the last visit (*p* < 0.05). The postoperative relative refractive error was − 0.13 ± 0.42 D (–0.60 D to + 0.57 D). The OCT showed that the IOLs were well centered, with a mean decentration of 0.20 ± 0.13 mm and a mean tilt of 2.31°±0.93°. Ten patients did not experience any complications.

**Conclusions:**

The modified Yamane technique in IOL fixation surgery, especially combined with iris reconstruction, reduces operation difficulty, increases operational stability and safety, and improves postoperative visual acuity without serious intra- or postoperative complications. The long-term improvement effect was remarkable.

**Supplementary Information:**

The online version contains supplementary material available at 10.1186/s12886-024-03493-8.

## Introduction

For intraocular lens (IOL) fixation in eyes lacking capsular or zonular support, the main clinical solutions include anterior chamber IOL, iris-fixated IOL, and scleral-fixated IOL [1–3]. Anterior chamber IOL fixation may lead to progressive corneal endothelial cell loss, pupil deformation, iris depigmentation, persistent inflammation, cystoid macular edema and secondary glaucoma. Therefore, this technique has rarely been used clinically [1, 3]. Iris-fixed IOLs require the use of a specially designed lens, which may not be available in some areas, and usually requires a structurally intact iris. In addition, these two types of IOLs are usually more expensive than regular posterior chamber IOLs. Therefore, scleral-fixated IOL implantation has become the most popular surgical method for aphakic eyes, especially for eyes with iris damage. Scleral-fixated IOL implantation surgeries vary in approach and can be categorized broadly into sutured IOL fixation and sutureless IOL fixation based on the specific surgical techniques applied.

In 2017, Yamane et al. proposed a sutureless double-needle technique using two 30-gauge thin-wall needles to assist the IOL haptics in tunneling through scleral and cauterize the externalized haptic ends to form flanges which are pushed back to the scleral tunnel for fixation. The Yamane technique is less invasive, reduces the risk of postoperative hypotony and infection, and avoids suture-related complications such as suture exposure, breakage, and knot erosion [4, 5].

However, in the initial phases of implementing the original Yamane technique in clinical practice, complications such as IOL haptic retraction, distortion, or breakage may arise from inadvertent mishandling, particularly when additional surgical procedures are involved. Furthermore, there is a paucity of long-term follow-up studies concerning the Yamane technique [6–8]. This report presents the clinical outcomes of employing a modified trailing-haptic-first approach within the Yamane technique, as well as the use of hand-made stoppers in surgeries that include combined iris reconstruction.

## Subjects and methods

This study complied with the *Helsinki Declaration* and was approved by the Ethics Committee of (blinded for review) Hospital. All patients provided written informed consent. The data of patients receiving IOL fixation with modified Yamane technique between December 2021 and August 2023 in the Ophthalmology Department of (blinded for review) Hospital were analyzed retrospectively. The longest follow-up duration was > 12 months.

General demographic information, preoperative and postoperative BCVA, corneal endothelial cells (CECs), IOP, OCT (Heidelberg Spectralis; Heidelberg Engineering, Heidelberg, Germany), fundus image, surgical indications and methods, intra- and postoperative complications were recorded. Anterior segment OCT (SS-ASOCT, CASIA2; TomeyCorp., Nagoya, Japan) was used to evaluate IOL tilt and decentration. The IOL Master 700 (Carl Zeiss Meditec, Jena, Germany) was used for ocular biometrics preoperatively, and the SRK/T or Barrett Universal II formula was used to calculate the IOL diopter. The inclusion criteria were aphakia, a dislocated IOL, a subluxated crystalline lens, and agreement with the study protocol. The exclusion criteria were severe eye disease requiring treatment, such as severe corneoscleral rupture, retinal detachment, optic neuropathy, choroidal rupture or macular pucker; IOP of 30 mmHg or more while receiving treatment with eye drops; scleritis; and postoperative follow-up for less than 3 months.

All surgeries were performed by the same experienced surgeon. The trailing-haptic-first modification of Yamane technique was used for intrascleral IOL fixation. Patients with lens or IOL dislocation underwent PPV combined with intrascleral IOL fixation. Patients with iridodialysis or mydriasis underwent iris reconstruction simultaneously. Hand-made haptic stoppers were used to temporarily stabilize the IOL during iris reconstruction.

### Surgical procedures

**Fig. 1:**
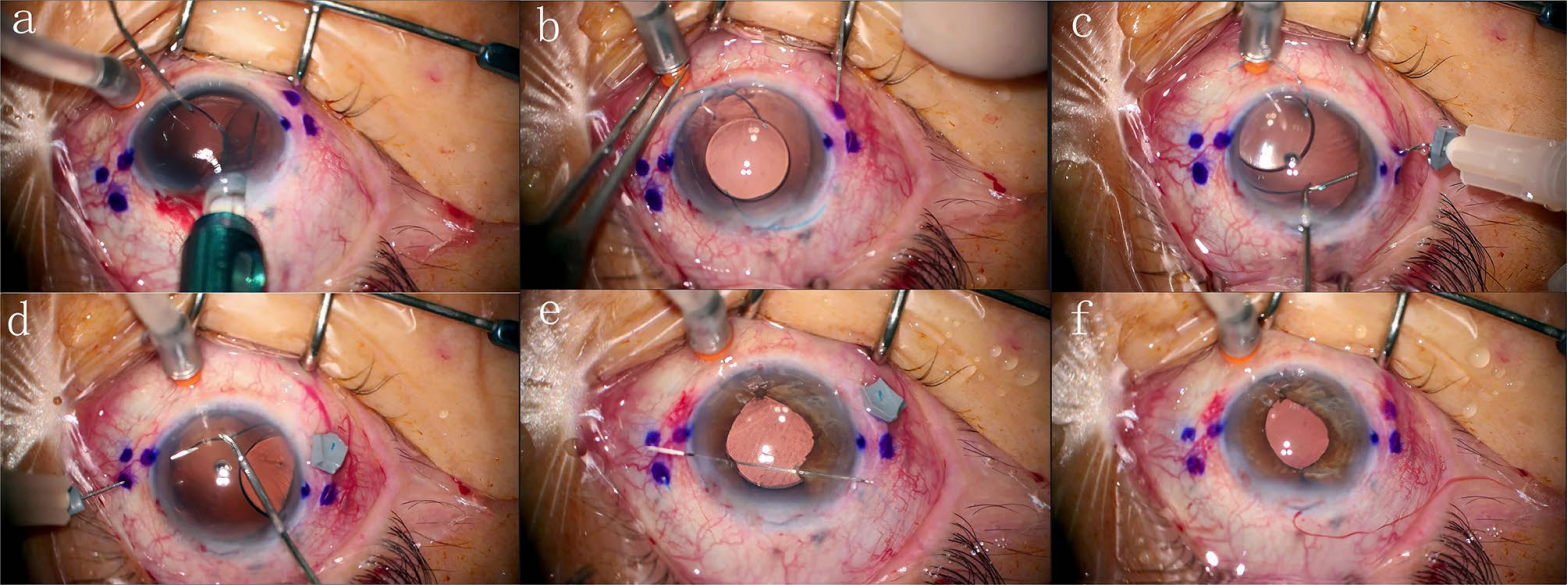
Modified Yamane technique combined with iris reconstruction. The 26-gauge needle is used to grasp the tip of the leading haptic through the inferior paracentesis (**a**); The leading haptic is externalized the inferior paracentesis, and the trailing haptic is left outside the superior incision (**b**); The trailing haptic is threaded into the lumen of the needle, externalized, and reinforced outside the eye (**c**); The leading haptic is threaded into the lumen of the needle, externalized, and reinforced outside the eye (**d**); Iris reconstruction is performed simultaneously after IOL fixation operation (**e**); The stoppers are removed, and the haptics are cauterized to form flanges and then pushed back into the sclera (**f**)

Haptic stoppers were made from the silicone cap of a viscoelastic syringe. The silicone cap was cut into approximately 2 mm × 2 mm pieces. The silicone piece was threaded onto the hub of the bending needle, then further trimmed to make it more compact and form a stopper. Inferior paracentesis and a superior 3 mm corneal incision were created. A 3-piece foldable IOL (Tecnis ZA9003, Abbott Medical Optics Inc., USA) was inserted into the chamber using an injector. A 26-gauge needle was placed through the inferior paracentesis to dock the leading haptic and externalize it, while the trailing haptic was left outside the superior incision. A right-sided angled sclerotomy was performed through the conjunctiva using a needle with a haptic stopper placed 2 mm from the limbal. The trailing haptic was threaded into the lumen of the needle and externalized with the needle. Following this, the silicone stopper was sleeved onto the haptic from the needle and adjusted to a suitable position. The leading haptic was externalized and reinforced similarly. Subsequently, additional manipulations, such as iris reconstruction, were performed. At the end of the surgery, the stoppers were removed, and the haptics were cauterized to form flanges. The haptics were then pushed back into the sclera. The procedure details were shown in Fig. 1. Besides, a surgery video has been included in the supplementary materials.

### Statistical analysis

The BCVA results were converted into logMAR values. The conversion correspondences of the index and visual acuities are as follows: index 2.0 logMAR, manual 2.3 logMAR, light perception 2.6 logMAR, and no light perception 2.9 logMAR. SPSS 22.0 software was used for statistical analysis. Measurement data was expressed by mean ± standard deviation. The Kolmogorov–Smirnov test was used to test data normality, the paired sample t-test or Wilcoxon rank sum test was used to test the results of the same index before and after the operation, and the chi-square test was used to classify variables. *p* < 0.05 was considered statistically significant.

## Results

The study included 12 patients (12 eyes). The detailed preoperative patient information was shown in Table 1. There were ten males and two females. Patients ranged in age from 54 to 79 years, with a mean age of 63 ± 10.61 years. The longest follow-up time exceeded 12 months. Of all the patients who received modified Yamane surgery, one (8.3%) had IOL dislocation, eight (66.7%) had lens dislocation or subluxation, and three (25.0%) had aphakic eyes. In the three aphakic eyes, traumatic lens dislocation was the main cause, PPV and lens extraction were performed in the primary surgery. Four of 12 patients underwent intrascleral IOL fixation combined with iris reconstruction. Details of the surgery were summarized in Table 2.

**Table 1:** Individual patient characteristics

Case	Age(years)	Gender	Operation indications	Adjuvant procedures	Previous operations	Follow-up time(months)
1	55	male	Aphakia	Iris reconstruction	PPV/Lens extraction/ILMP	6
2	56	male	Crystalline lens subluxation	PPV/Lens extraction	None	8
3	79	male	Crystalline lens subluxation	PPV/Lens extraction/Iris reconstruction	None	7
4	77	male	Crystalline lens subluxation	PPV/Lens extraction	None	6
5	63	female	Crystalline lens subluxation	PPV/Lens extraction	None	6
6	53	female	Aphakia	None	PPV/Lens extraction/ERMP/Endolaser	7
7	57	male	Crystalline lens subluxation	PPV/Lens extraction/Iris reconstruction	None	6
8	54	male	Aphakia	Iris reconstruction	PPV/Lens extraction/Iris reconstruction/Cyclopexy/Endolaser	12
9	59	male	Crystalline lens dislocation	PPV/Lens extraction	None	14
10	86	male	Crystalline lens dislocation	PPV/Lens extraction	None	11
11	58	male	Crystalline lens dislocation	PPV/Lens extraction	None	13
12	63	male	IOL dislocation	PPV/IOL removal	None	3

**Table 2:** Operative details

Operative details	
Lens type	ZA9003
Lens power	+ 12.5 - +24.0D
Combined ocular procedures and Surgery	
PPV	9(75.0%)
Complex lens extraction	8(66.7%)
Iris reconstruction	4(33.3%)
IOL removal	1(8.3%)

**Fig. 2:**
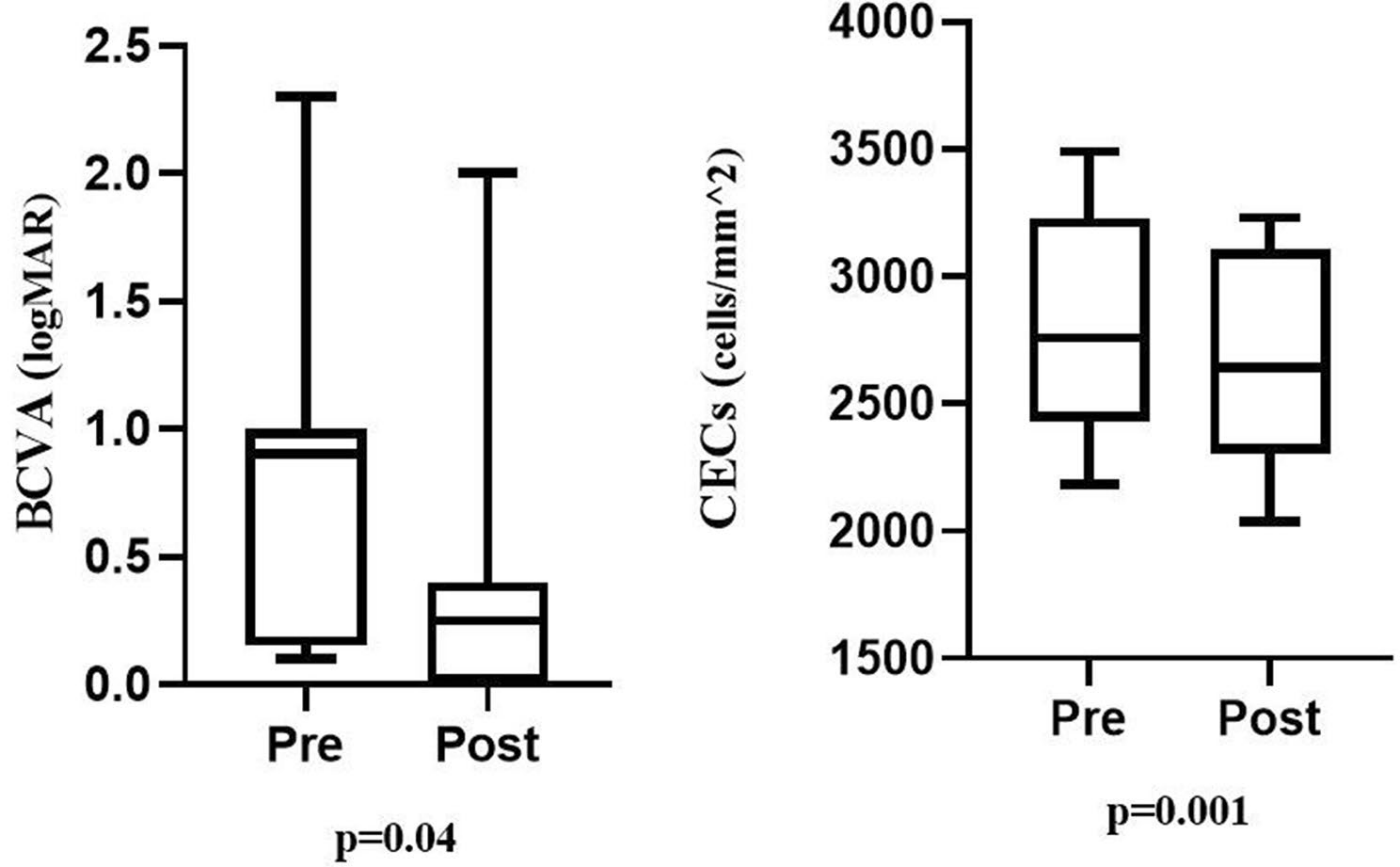
Comparison of preoperative and postoperative BCVA and CECs

As shown in Table 3, the mean BCVA (0–2.0 logMAR) of the participants was 0.44 ± 0.53 logMAR one week after surgery and 0.39 ± 0.56 logMAR at the last visit. Postoperative short-term and long-term mean BCVA were significantly improved compared with preoperative (0.89 ± 0.72 logMAR) Fig. 2. Of 12 patients, 11 (91.7%) showed improved visual acuity. During the long-term follow-up, the IOP of all patients remained in normal range (15.3 ± 3.3 mmHg). The CECs of all patients were within the normal range (2632 ± 416 cells/mm2). The mean predicted refractive power before surgery was − 0.63 ± 1.01 D (–3.00 D to 0 D), and the actual postoperative equivalent spherical power was − 0.75 ± 1.03 D (–3.1 D to + 0.5 D). The mean refractive difference was − 0.13 ± 0.42 D (–0.60 D to + 0.57 D).

**Table 3:** Clinical data in preoperative and postoperative period

Case	BCVA (logMAR)			IOP	CECs, cells/mm^2		Complications
	Pre	Post (1 week)	Post (last visit)	Post	Pre	Post	
1	1.00	2.00	2.00	19	2701	2601	None
2	1.00	0.30	0.30	16	2815	2679	None
3	2.30	1.00	1.00	13.3	2240	2045	None
4	1.00	0.40	0.40	14	2185	2037	None
5	0.70	0.40	0.30	15.3	3462	3230	None
6	0.30	0.10	0.00	12.6	3234	3105	None
7	0.10	0.20	0.00	9	2449	2330	None
8	0.80	0.30	0.20	19.9	2869	2699	Transient IOP elevation
9	0.10	0.00	0.00	18.6	3490	3155	None
10	2.30	0.20	0.10	19.6	3209	3102	Macular edema
11	0.10	0.00	0.00	11.7	2426	2301	None
12	1.00	0.40	0.40	14.1	2556	2310	None
Mean ± SD	0.89 ± 0.72	0.44 ± 0.53	0.39 ± 0.56	15.3 ± 3.3	2803 ± 437	2632 ± 416	

During the follow-up, one patient (8.3%) had transiently elevated IOP one week after the surgery, which returned to normal after topical medication. One patient (8.3%) developed macular edema half a year after surgery, which was resolved spontaneously at the last visit. No serious complications, such as IOL displacement, ocular hypotony, corneal decompensation, retinal detachment, choroidal detachment, or endophthalmitis, were found in any patient during or after the surgery. At the last visit, anterior segment OCT showed that the IOLs were well-centered without apparent tilt or decentration, with an average tilt of 2.31°±0.93° and an average decentration of 0.20 ± 0.13 mm (Table 4).

**Table 4:** Parameters of intraocular lens position at the last visit

Case	Tilt, degree	Decentration, mm
1	3.5	0.54
2	2.1	0.21
3	4.2	0.33
4	2.8	0.18
5	1.8	0.13
6	1.2	0.05
7	1.5	0.13
8	2.6	0.21
9	1.6	0.12
10	1.3	0.06
11	1.8	0.13
12	3.3	0.32
Mean ± SD	2.31 ± 0.93	0.20 ± 0.13

## Discussion

This study retrospectively analyzed the long-term outcomes of 12 patients (12 eyes) using trailing-haptic-first modification of Yamane technique for intrascleral IOL fixation combined with or without iris reconstruction. Long-term follow-up revealed that most patients (91.7%) showed stable improvement in visual acuity. Although 91.7% of patients underwent additional surgical procedures simultaneously, the incidence of postoperative complications was low. Only one patient experienced a transient increase in intraocular pressure within 1 week after surgery, and another patient developed macular edema half a year after surgery, which later resolved spontaneously. During the follow-up period, no significant IOL tilt or decentration occurred in any patient.

Since Gabor et al. pioneered the application of sutureless intrascleral IOL fixation technique in 2007, this technique has been increasingly used in secondary IOL implantation in eyes lacking capsular or zonular support caused by various reasons [6]. In 2017, Yamane et al. proposed a flanged intrascleral IOL fixation with double-needle technique, in which way two 30-gauge thin-wall needles were used to externalize the IOL haptics simultaneously, then the haptic ends were cauterized to form flanges which were finally pushed back to the scleral tunnel for IOL fixation [4].

The double-needle technique, while seemingly straightforward, presents considerable challenges to novices in the field. Many surgeons are still accustomed to thread two haptics of the IOL sequentially. The externalization of the leading haptic constrains the IOL’s mobility, which can result in complications such as haptic kinking, fracture, and slippage while threading the trailing haptic into the needle. Ocular trauma is one of the main causes of insufficient capsular or zonular support, with a significant number of patients sustaining injuries to anterior segment tissues, including iridodialysis and pupil paralysis. For these patients, additional surgical manipulations, such as iris reconstruction, are often required simultaneously with the IOL fixation.

The Yamane technique’s reliance on the resistance provided by the scleral tunnel and the flanged haptic ends for IOL fixation may result in suboptimal stability prior to the formation of a scleral scar. Therefore, any further manipulations of the anterior segment could potentially lead to the retraction of the IOL haptics. Moreover, in regions where 30-gauge thin-wall needles are not available, such as in China, practitioners commonly use 26-gauge needles as an alternative. The standard, non-thin-walled 30-gauge needles, with their narrower inner diameter, do not always allow for the smooth insertion of the IOL haptics. On the other hand, the scleral tunnel created by a 26-gauge needle may be relatively more spacious in comparison to the IOL haptics, which heightens the risk of haptic retraction during subsequent anterior segment procedures.

A modified Yamane technique was used in our study. The most considerable improvement in this surgical technique is the trailing-haptic-first approach, which prevents distortion and potential fracture of the trailing haptic stemming from the surgeon’s technical constraints during the double-needle operation [7]. The step-by-step operation bypasses the intraoperative problems described before and provides a safer and easier approach for the surgeon. Additionally, traumatic lens or IOL dislocation was observed in 10 of the 12 patients, with four required combined anterior segment repair surgery. We employed haptic stoppers fashioned from the silicone caps of viscoelastic syringes to temporarily secure the IOL haptics. This measure prevented haptic retraction amid additional anterior segment surgeries, thus improving overall surgical stability and safety [8]. The sterile silicone material used is both readily accessible and simple to craft in the operating room, without incurring additional costs. Ucar F et al. have reported that the flattened flanged IOL fixation technique with 27G needles in intraocular lens implantation provided effective IOL fixation, with firm haptic fixation, without use of sutures or glue [9]. However, the scleral tunnel made by a 26G needle is more spacious, and we have no more practical experience on to ascertain whether this technique could enhance the stability of the IOL in surgeries combined with iris reconstruction. Among the 11 patients who received combined IOL fixation and other procedures, such as PPV and iris reconstruction, there were no significant intraoperative or postoperative complications reported over the long-term follow-up. Moreover, the incidence of complications was far lower than those reported in earlier studies [4, 5, 10–13]. The mean postoperative BCVA was significantly improved compared with that before surgery (0.39 ± 0.56 logMAR vs. 0.89 ± 0.72 logMAR, *p* < 0.05). At the final follow-up, the mean BCVA was comparable to the findings of Vural et al., demonstrating an increase to − 0.37 ± 0.27 logMAR [13–17]. However, considering the differences in study design, inclusion criteria, and notably, the significantly smaller sample size, it seems not feasible to directly compare our result in this study with other.

There have been few statistical reports on postoperative IOL tilt. Yamane et al. observed an average IOL tilt of 3.4°±2.5°, while Kumar et al. reported a mean tilt of 3.2°±2.7° [4, 5, 18]. In our study, the mean IOL tilt at the final follow-up was 2.31°±0.93°. Given that cases undergoing scleral tunnel fixation of the IOL often involve additional intraocular structural abnormalities and due to the small sample size, it is difficult to make a direct comparison with results from different studies.

The precise calculation of preoperative IOL power is critical, yet prior research indicates that postoperative refractive outcomes often bear a degree of unpredictability, with postoperative refractive states ranging from mild hyperopia to mild myopia. Retrospective analyses of sutureless intrascleral IOL fixation have yielded varied results; some studies report a myopic shift, while others indicate a hyperopic shift [4, 5, 10, 12–14, 19–21]. In our study, the mean refractive difference from predicted value showed mild myopic shift (− 0.13 ± 0.42 D), aligning with the findings of Yamane et al. Within our patient cohort, 63.6% exhibited a myopic shift, 36.4% a hyperopic shift, and notably, 63.6% achieved a target refractive error within 0.5 D.

To date, no single surgical method has been shown to completely prevent postoperative refractive shift. Variations in refractive results are related to several factors, including choice of IOL, calculation formula, and fixation methods, such as the haptic angle and scleral tunnel length [4, 14]. This study used a modified Yamane technique and hand-made silicone stoppers to increase surgical stability. The refractive error of 63.6% of patients was < 0.5 D, which greatly improved the postoperative visual effect of patients. However, further research is needed to explore more effective surgical methods to prevent refractive shift.

Limitations of this study are the small sample size, limited follow-up time, and lack of comparison with other IOL fixation method. In future, more samples and longer follow-up, as well as controlled prospective studies, are needed to provide more reliable evidences for the safety and effectiveness of this modified Yamane technique.

In conclusion, the trailing-haptic-first modification of the Yamane technique provides an alternative to secondary IOL implantation in eyes lacking capsular or zonular support. The utilization of hand-made silicone stoppers can temporarily reinforce the IOL when additional surgical manipulations are combined, such as iris reconstruction. This method proves to contribute to stability and reliability, not only enhancing patient visual outcomes and reducing intraoperative and postoperative complications, but also facilitating a shorter learning curve for surgeons. To further validate and endorse this modified technique, studies involving larger patient cohorts and extended follow-up periods are warranted.

### Electronic supplementary material

Below is the link to the electronic supplementary material.

Supplementary Material 1

## Data Availability

The data that support the findings of this study are available from the corresponding author upon reasonable request.
